# Supplementary material for: A whole-genome scan for evidence of positive and balancing selection in aye-ayes (Daubentonia madagascariensis) utilizing a well-fit evolutionary baseline model
Source: G3 (Bethesda). 2025 Apr 10;15(7):jkaf078. doi: 10.1093/g3journal/jkaf078 (PMC12239616; doi:10.1093/g3journal/jkaf078)
Supplement: jkaf078_Supplementary_Data [file jkaf078_supplementary_data.zip › Supplementary_Tables_G3-2025-405835.pdf]

SUPPLEMENTARY TABLES

	NCBI	coverage
	DMad_01	104.9
	DMad_02	50.5
	DMad_03	50.2
	DMad_04	53.7
	DMad_05	52.5

Supplementary Table S1. Samples and their sequencing coverage.

scaffold	# SNPs	# invariant sites	Ts/Tv
1	310,744	202,589,728	2.49
2	282,464	187,007,285	2.53
3	266,594	166,053,610	2.46
4	221,453	141,877,129	2.51
5	213,515	139,978,054	2.57
6	201,488	134,246,862	2.63
7	197,047	125,387,556	2.56
8	160,561	106,567,069	2.56
10	111,122	74,651,313	2.66
11	101,805	68,490,964	2.88
12	68,829	42,837,589	2.89
13	66,853	39,350,940	2.84
14	42,375	22,947,744	2.77
15	36,095	19,884,749	2.76
$\sum$ or $\varnothing$	**2,280,945**	**1,471,870,592**	**2.60**

Supplementary Table S2. Summary of the variant and invariant sites. After filtering, a total of >2.2 million autosomal, biallelic, single nucleotide polymorphisms (SNPs) with a transition-transversion ratio (Ts/Tv) of 2.60 were discovered in the accessible genome.

	mapping confidence (%)				
**gene**	**DMad_01**	**DMad_02**	**DMad_03**	**DMad_04**	**DMad_05**
OR2M4	99.999900	99.999900	99.999898	99.999900	99.999900
OR4A16	99.628465	25.698086	99.542912	99.698005	99.275564
OR5AC2	99.999900	99.999900	99.999900	99.999900	99.999895
OR5H2	99.999712	99.998415	99.999852	99.999611	99.999852
OR5H6	99.999766	99.999669	99.999898	99.999830	99.999900
OR5H14	99.999893	99.999900	99.999898	99.999898	99.999900
OR6C74	99.999898	99.999898	99.999895	99.999893	99.999898
OR8U3	99.999900	99.999895	99.999898	99.999900	99.999900
OR10A7	99.999900	99.999900	99.999898	99.999900	99.999900
OR10G8	99.999743	99.999224	99.999206	99.999669	99.999354
OR10G9	99.999149	99.999425	99.999276	99.999369	99.998928

Supplementary Table S3. Confidence scores of read mappings in the OR genes meeting the null thresholds for selective sweeps and balancing selection per individual. Values shown in red are below the benchmarked high-confidence threshold.
